# Comparison of Patient-Reported vs Physician-Estimated Angina in Patients Undergoing Elective and Urgent Percutaneous Coronary Intervention

**DOI:** 10.1001/jamanetworkopen.2020.7406

**Published:** 2020-06-19

**Authors:** John T. Saxon, Paul S. Chan, Andy T. Tran, Suveen Angraal, Phillip G. Jones, J. Aaron Grantham, John A. Spertus

**Affiliations:** 1 St Luke’s Mid America Heart Institute, Kansas City, Missouri; 2 University of Missouri-Kansas City, Kansas City, Missouri

## Abstract

This cohort study assesses the use of patient-reported vs physician-estimated angina in patients undergoing percutaneous coronary intervention.

## Introduction

Coronary revascularization in stable ischemic heart disease (SIHD) is indicated for angina relief.^1,2^ In practice, the Canadian Cardiovascular Society (CCS) classification is used to quantify angina. However, this classification is a physician’s estimate of angina rather than a patient-reported measure, and there is evidence of discordance between these measures.^3^ Whether discordance between physician-assessed and patent-reported angina exists in patients undergoing percutaneous coronary intervention (PCI) is unclear. Therefore, we compared these measures in a cohort of patients undergoing PCI.

## Methods

This cohort study was conducted in accordance with the Strengthening the Reporting of Observational Studies in Epidemiology (STROBE) reporting guideline. The institutional review board of Saint Luke’s Mid America Heart Institute approved the study, and the institutional review boards of the participating medical centers (eAppendix in the Supplement) waived the requirement for patient written informed consent because this study’s analyses were done within a study to improve the process of informed consent; this study was deemed a quality improvement initiative. Data were collected from April 13, 2009, to October 31, 2011, and were analyzed from November 20, 2019, to March 4, 2020.

We used the Seattle Angina Questionnaire (SAQ) to collect patient-reported angina and the CCS classification for physicians’ estimates of angina from 10 medical centers that were part of the Patient Risk information Services Manager study between 2009 and 2011 (eAppendix in the Supplement). The CCS class was obtained from the National Cardiovascular Data Registry record. The SAQ angina frequency (AF) domain (scores of 0-30, 31-60, 61-99, and 100 representing daily, weekly, monthly, and no symptoms of angina, respectively) was used to assess the burden and frequency of angina over the previous 4 weeks (eAppendix in the Supplement) and the score correlates closely with daily angina diaries.^4^ The CCS classification was used to stratify angina into levels 0 to IV, with higher classes corresponding with increasing symptom burden.^5^

## Results

This cohort study included 759 patients who underwent PCI for SIHD, and 895 who underwent PCI for unstable angina. Mean (SD) patient age was 64.3 (10.7) years, 71% were men, 44.4% had a prior PCI, and 21.7% had a prior coronary artery bypass grafting procedure. For patients treated for SIHD, 267 of 759 (35.2%) had an SAQ-AF score of 100 (no angina). Of these patients, 33 (12.4%) and 20 (7.5%) were classified by physicians as having moderate (CCS class II) or severe (CCS class III-IV) angina, respectively (Table, Figure). For patients with unstable angina, 110 of 895 (12.3%) had an SAQ AF of 100. Of these patients, 12 (10.9%) were categorized by physicians as having moderate symptoms, and 39 patients (35.5%) were categorized has having severe symptoms (Table, Figure).

**Table.** CCS Classification vs SAQ AF Scores in Patients Undergoing Percutaneous Coronary Intervention for Stable Ischemic Heart Disease and Unstable Angina

Cohort and CCS class	No. of patients and SAQ AF score				
	0-30 (Daily)	31-60 (Weekly)	61-99 (Monthly)	100 (No angina)	Total
**Stable angina cohort**					
IV	22	30	27	8	87
III	20	51	38	12	121
II	14	89	101	33	237
I	3	16	36	17	72
0	1	15	29	197	242
Total No. (%)	60 (8.0)	201 (26.5)	231 (30.4)	267 (35.2)	759
**Unstable angina cohort**					
IV	46	118	86	21	271
III	34	124	99	18	275
II	22	78	98	12	210
I	4	20	25	7	56
0	2	14	15	52	83
Total No. (%)	108 (12.1)	354 (39.6)	323 (36.1)	110 (12.3)	895

Abbreviations: CCS, Canadian Cardiovascular Society; SAQ AF, Seattle Angina Questionnaire Angina Frequency.

**Figure.**
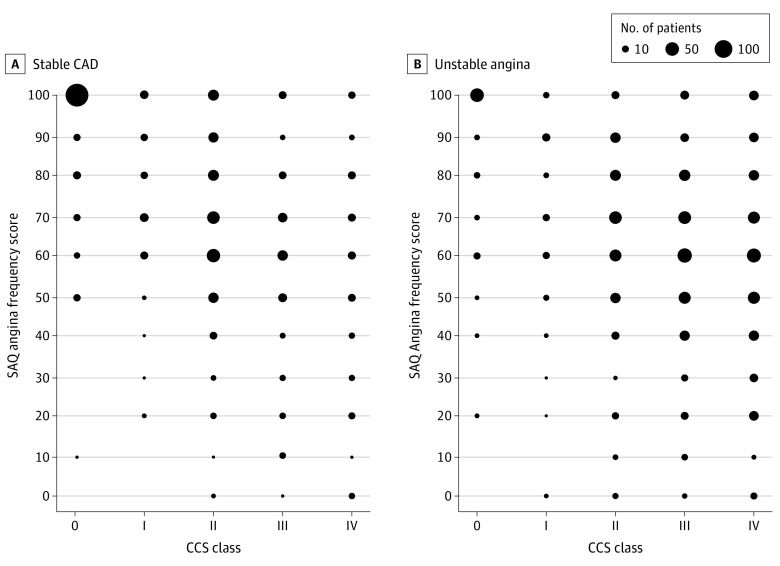
Physician-Estimated Canadian Cardiovascular Society (CCS) Class vs Seattle Angina Questionnaire (SAQ) Angina Frequency Scores in Patients Undergoing Percutaneous Coronary Intervention for Stable Ischemic Heart Disease and Unstable Angina CAD indicates coronary artery disease.

## Discussion

Although validated patient-reported instruments are available, angina assessment in clinical practice relies on physicians’ estimates. We found that 35.5% of patients undergoing PCI for SIHD and 12.3% of those with unstable angina reported no angina within 4 weeks. However, many of these patients were categorized by physicians as having moderate or severe symptoms. These findings build on studies showing discordance between assessments of health status^3^ and suggest that patients’ reports of angina symptoms may be more relevant than physician’s estimates. As new data emerge from studies such as ISCHEMIA^6^ and highlight the limited benefits of revascularization in asymptomatic patients, it is increasingly important to directly elicit patients’ experiences.

This study has limitations. First, data were collected 9 years ago, although there is no reason to expect patterns of angina assessment would have changed substantially over time. Second, patients with unstable angina typically have recent symptom onset (eg, within 24 hours), and the SAQ is designed to assess symptoms over the last 4 weeks. Third, physicians’ estimates of unstable angina may also be inaccurate in an era of Appropriate Use Criteria in which higher ratings for are assigned for unstable angina than for SIHD. Fourth, we cannot exclude the possibility that some patients had atypical presentations that were not captured by the SAQ; whether other instruments, including the Rose Dyspnea Score, may be more sensitive to atypical angina is unknown. In addition, we did not assess angina at follow-up; thus, conclusions regarding the best method of assessment at baseline could not be drawn from the context of this study.

In this cohort study, 35.5% of patients undergoing PCI for SIHD and 12.3% of those undergoing PCI for unstable angina self-reported no angina. Among patients without self-reported angina, 1 in 5 undergoing elective PCI and 1 in 2 undergoing urgent PCI were assessed by physicians to have moderate-to-severe angina. These data have important implications for patient selection for coronary revascularization.

## Notes

Supplement. **eAppendix****.**
