# Supplementary material for: Comparison of Patient-Reported vs Physician-Estimated Angina in Patients Undergoing Elective and Urgent Percutaneous Coronary Intervention
Source: JAMA Netw Open. 2020 Jun 19;3(6):e207406. doi: 10.1001/jamanetworkopen.2020.7406 (PMC7305522; doi:10.1001/jamanetworkopen.2020.7406)
Supplement: Supplement. — eAppendix. [file jamanetwopen-3-e207406-s001.pdf]

Supplementary Online Content

Saxon JT, Chan PS, Tran AT, et al. Comparison of patient-reported vs physician-estimated angina in patients undergoing elective and urgent percutaneous coronary intervention. *JAMA Netw Open*. 2020;3(6):e207406. doi:10.1001/jamanetworkopen.2020.7406

eAppendix.

This supplementary material has been provided by the authors to give readers additional information about their work.

© 2020 Saxon JT et al. *JAMA Network Open.*

eAppendix

Information regarding ePRISM:

The study was approved by the institutional review board at each center, and patients provided informed consent to participate. The analysis for this study was conducted 2019-2020. The study adheres to the Strengthening the Reporting of Observational Studies in Epidemiology (STROBE) guideline.

Centers participating in the Patient Risk information Services Manager (ePRISM) where patients were enrolled and data collected:

1) Washington University, St. Louis, MO
2) Integris Hospital, Oklahoma, OK
3) Yale University, New Haven, CT
4) Henry Ford Hospital, Detroit, MI
5) Baystate Medical Center, Springfield, MA
6) The Heart Hospital at Baylor, Plano, TX
7) Kaiser Hospital, San Francisco, CA
8) St. John's Hospital, Springfield, IL
9) Mayo Clinic, Rochester, MN
10) Saint Luke's Mid America Heart Institute, Kansas City, MO

Patients enrolled in ePRISM:

3,299 patients were enrolled between 2009 – 2011. 1,061 were not included in this study due to indications for PCI other than stable ischemic heart disease and unstable angina: 651 for NSTEMI; 76 for STEMI; 195 for staged PCI; 70 for congestive heart failure; 35 for arrhythmia management; and 34 for pre-operative optimization prior to non-cardiac surgery. Of the 2,238 patients in whom PCI was performed, 4 patients were exluded due to missing baseline health status, and 580 did not have documentations of CCS class, leaving 1,654 for the final study cohort (750 for stable ischemic heart disease and 895 for unstable angina).

Information regarding the Seattle Angina Questionnaire:

The SAQ is a validated 19-item questionnaire assessing patients' frequency of angina as well as angina-related physical limitation, treatment satisfaction, and quality of life. We focused on the SAQ angina frequency (SAQ AF) domain, which assesses the burden and frequency of angina symptoms over the previous 4 weeks and has been shown to correlate closely with daily angina diaries. The SAQ AF domain is scored on a 100-point scale; where a score of 100 indicates no angina over the past 4 weeks and scores of 0-30, 31-60, and 61-99 correspond to daily, weekly, and monthly angina respectively.

Information regarding the CCSC:

© 2020 Saxon JT et al. *JAMA Network Open.*

The CCSC stratifies angina into 5 levels, with CCSC 0 = no symptoms, CCSC I = symptoms only with strenuous exertion, CCSC II = symptoms only with moderate exertion, CCSC III = symptoms with mild exertion, and CCSC IV = angina at rest.

© 2020 Saxon JT et al. *JAMA Network Open.*
